# Computational systems-biology approaches for modeling gene networks driving epithelial–mesenchymal transitions

**DOI:** 10.1002/cso2.1021

**Published:** 2021-06-09

**Authors:** Ataur Katebi, Daniel Ramirez, Mingyang Lu

**Affiliations:** 1 Department of Bioengineering, Northeastern University, Boston, Massachusetts, USA; 2 Center for Theoretical Biological Physics, Northeastern University, Boston, Massachusetts, USA; 3 College of Health Solutions, Arizona State University, Tempe, Arizona, USA

**Keywords:** bottom-up approach, epithelial-mesenchymal transition, gene regulatory network, network construction, network modeling, top-down approach

## Abstract

Epithelial–mesenchymal transition (EMT) is an important biological process through which epithelial cells undergo phenotypic transitions to mesenchymal cells by losing cell–cell adhesion and gaining migratory properties that cells use in embryogenesis, wound healing, and cancer metastasis. An important research topic is to identify the underlying gene regulatory networks (GRNs) governing the decision making of EMT and develop predictive models based on the GRNs. The advent of recent genomic technology, such as single-cell RNA sequencing, has opened new opportunities to improve our understanding about the dynamical controls of EMT. In this article, we review three major types of computational and mathematical approaches and methods for inferring and modeling GRNs driving EMT. We emphasize (1) the bottom-up approaches, where GRNs are constructed through literature search; (2) the top-down approaches, where GRNs are derived from genome-wide sequencing data; (3) the combined top-down and bottom-up approaches, where EMT GRNs are constructed and simulated by integrating bioinformatics and mathematical modeling. We discuss the methodologies and applications of each approach and the available resources for these studies.

## 1 INTRODUCTION

Epithelial–mesenchymal transition (EMT) is an important cellular process, during which epithelial cells (E) convert to mesenchymal cells (M) by changing their morphology from cobblestone shape to spindle shape, losing tight cell–cell adhesion, and gaining motility and invasiveness [1, 2]. EMT and its reverse process, mesenchymal–epithelial transition (MET), have been shown to play a crucial role in multiple biological phenomena, such as embryonic development, wound healing, and cancer metastasis [3]. It is worth noting that recent studies have identified a spectrum of hybrid EMT states, featuring the coexistence of both E and M traits [4, 5]. In a hybrid state, cells retain cell–cell adhesion and meanwhile become motile, thus allowing collective cell migration, a phenomenon related to cancer invasiveness [6].

**FIGURE 1:**
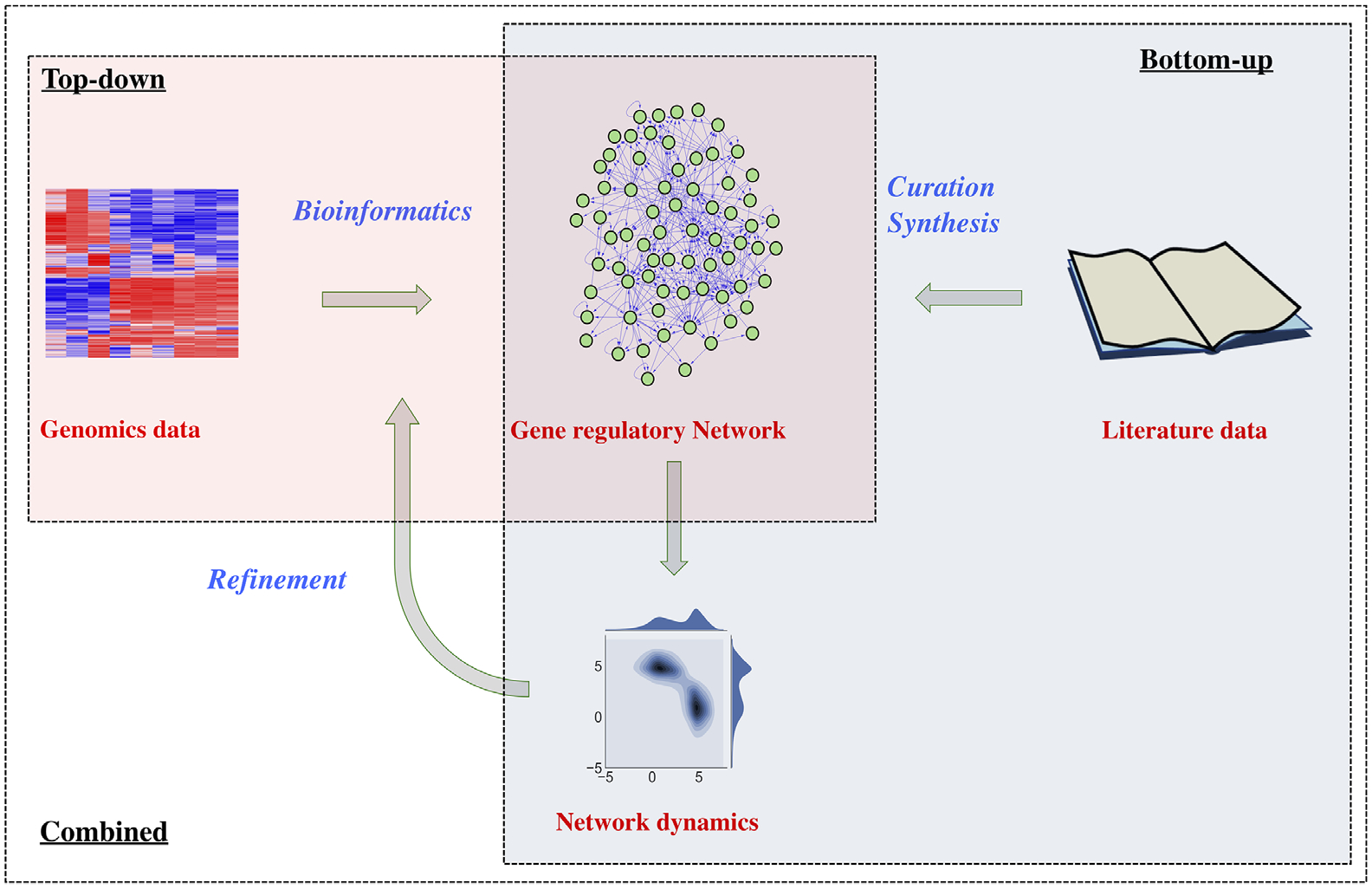
Graphical summary of the three broad approaches discussed in this review. (A) Bottom-up approaches (blue shaded box): manual literature review or database search is used to construct a GRN, which is then modeled with mathematical modeling to elucidate the network gene expression dynamics and the property of network control. GRNs are usually constructed based on simulation outcomes. (B) Top-down approaches (red shaded box): algorithmic and statistical tools are used to infer GRNs from various omics assays of a dataset of interest. GRNs are usually constructed based on statistical test. (C) Integrated top-down and bottom-up approaches (outermost box): these studies combine mathematical modeling, with an emphasis on the system dynamics, with high-throughput bioinformatics assays to construct GRNs that agree with both specific experimental observations and general understanding of EMT dynamics

To understand the properties of the EMT-related state transitions, many experimental and computational studies have been undertaken to elucidate the gene regulatory mechanisms driving EMT. In particular, substantial efforts have been made with computational systems biology approaches to model EMT gene regulatory networks (GRNs). A high-quality GRN model can enhance our understanding of the molecular drivers of EMT, the relationship between various EMT states, and the coupling of EMT with other biological processes. GRN models also allow us to generate new predictions, such as the outcomes of gene knockdown, which lead to testable hypotheses for new experimental studies. So far, the existing network modeling studies can be categorized into three types: (1) the bottom-up approach, where GRNs are derived from the analysis and synthesis of literature data, followed by mathematical modeling for network dynamics simulations; (2) the top-down approach, where GRNs are derived from genomics data, such as gene expression, by bioinformatics methods featuring statistical analysis; (3) a more recent methodology that integrates both the bottom-up and top-down approaches, typically involving both bioinformatics and network simulations (Figure 1). Here, we will explain and review these types of computational and theoretical studies on EMT GRN modeling. For each approach, we will discuss the methodology and its applications and the currently available resources for its studies.

## 2 THE BOTTOM-UP APPROACH

The most common and popular approach for modeling EMT GRNs relies on an extensive literature search for biological evidence of gene regulatory interactions, from which researchers assemble a gene network. Mathematical modeling is then applied to the constructed GRNs to evaluate their gene expression dynamics. A good GRN model can not only capture the essential dynamical behavior of a biological system, but also provides new testable predictions for experimental validation, shedding new insights and permitting a deeper understanding of the system. Due to extensive previous studies on EMT [7, 8], abundant biological evidence for gene regulatory interactions during EMT is available, particularly in the area of cancer research [9, 10] and developmental biology [11]. These experimental studies have led to some successful modeling efforts on EMT GRNs [12–14], where literature-based GRNs were simulated to elucidate the heterogeneity of EMT states and the control mechanism of the cellular state transitions between them. These simulation studies have generated new predictions, which can then be tested experimentally.

GRN models in the bottom-up approach can be of three categories: those that focus on a core gene regulatory circuit of EMT master regulators, those that model a large GRN of detailed gene regulators and/or upstream signaling pathways, and those that investigate the coupling of EMT circuit with circuits of other biological processes. In the following, we will describe the research efforts in those directions.

### 2.1 Small EMT circuits

In a typical study using the bottom-up approach, one synthesizes the literature data to construct a small circuit model, from which one elucidates its regulatory mechanism. Some early modeling studies on EMT GRNs focused on core gene regulatory circuits, consisting of the EMT master regulators: two microRNA families miR34 and miR200 and two transcription factor families ZEB and SNAIL [15, 16]. These models incorporated signaling nodes such as transforming growth factor beta (TGF-*β*) to drive the circuits and some targeted genes such as CDH1 and VIM as circuit readout. Because of the essential role of microRNAs in the translational regulation of key transcription factors [17], new mathematical formalisms were introduced [15, 18] to model microRNA-mediated translational inhibition and mRNA degradation. A typical way to model a GRN is to first write down the chemical rate equations (typically ordinary differential equations) and then apply nonlinear dynamics methods, such as nullcline and bifurcation, to identify the possible stable steady states of the GRN. These ordinary differential equation (ODE)-based modeling studies predicted not only the epithelial (E) and mesenchymal (M) states, but also a hybrid state (E/M) with features of both epithelial and mesenchymal phenotypes. The predicted hybrid E/M state was later identified experimentally [6], and its important role was found in tumorigenesis [19, 20]. Core EMT circuit models have also been carefully evaluated [21] and validated experimentally, for example, by flow cytometry measurement of E-cadherin and vimentin in TGF-*β*1-induced EMT of MCF10A cell line [22]. Furthermore, energy landscape analysis has been applied to the core EMT circuit, from which access to a hybrid state was shown to depend on the extracellular environment [23].

These circuit models have further been extended to incorporate additional genes, such as OVOL2, GRHL2, Np63*α*, NFATc, and NRF2, with a research focus on their role in stabilizing/destabilizing the hybrid state in cancer metastasis [18, 24–28]. Recently, Celia-Terrassa et al. characterized two distinct types of EMT dynamics (hysteretic and nonhysteretic) through their ODE/PDE-based modeling of a small EMT circuit (TGF-*β*, Zeb1/2, miR-200, and E-cadherin) and identified their association with metastasis and clinical outcomes using mouse models [29].

Overall, these studies of the EMT circuits demonstrate the usefulness of investigating small circuit models, typically constructed based on expert knowledge in the EMT literature. Mathematical modeling of these small EMT circuits sheds light on a mechanistic understanding of EMT. However, in some cases certain regulators of interest may not be captured by a small EMT circuit, therefore researchers are also interested in constructing and modeling larger EMT GRNs.

### 2.2 Large EMT networks

Construction of large EMT GRNs relies on more extensive literature search, typically incorporating (1) more detailed gene components, including factors from the same gene families, (2) signaling pathways upstream to EMT master regulators, and (3) in some cases, readout nodes representing the downstream EMT-related processes.

In particular, Steinway et al. synthesized existing literature data and constructed a 70-node EMT network representing the conserved regulation of EMT [30]. They gathered interactions primarily from hepatocellular carcinoma (HCC) EMT and secondarily from other tissue types, which produced an EMT GRN incorporating different molecular processes involving growth factors, signal transduction pathways, and transcription regulators. They simulated the GRN using a Boolean network model to understand the signaling abnormalities in the HCC progression [30] and their implication of combinatorial therapy by gene perturbation [31]. Here, Boolean network models describe the node status (gene expression or activity of a biological process) with two discrete values (i.e., 0 and 1) and simulate the network dynamics by updating the node status using Boolean functions [32]. Font-Clos et al. extended the GRN to a 72-node network and performed Boolean network modeling to construct a topographic map [33]. They studied the phenotypic stability of the topographic landscape using Ising model, where they identified a series of metastable hybrid EMT states, a prediction that is supported by RNA-seq data from both lung adenocarcinoma and embryonic differentiation. In a recent study, Silveira et al. constructed an 18-node literature-based EMT GRN to simulate EMT using Boolean network modeling [34]. In addition, some researchers augmented the literature-based approach by incorporating bioinformatics methods to construct larger EMT networks [35, 36] (details in Section 6).

Besides, Huang et al. [37] extended a core EMT circuit [15, 16] to a 22-node GRN by incorporating EMT factors from Ingenuity Pathway Analysis [38] and additional literature data [30, 31, 39, 40]. Instead of using Boolean network modeling as in many other large network studies, they devised a modeling method named random circuit perturbation (RACIPE), an ODE-based modeling method to generate an ensemble of kinetic models corresponding to a fixed GRN topology. Because RACIPE allows to model the time dynamics of continuous gene expression levels, it can better capture the intermediate levels of gene expression and is more effective to characterize hybrid states of a GRN, as supported by a recent study that compared RACIPE and Boolean simulations for various EMT GRNs of different sizes [41]. Also, with the RACIPE framework, Kohar and Lu showed that stochasticity in gene regulation and cell-to-cell variability can stabilize these hybrid EMT states [42].

In summary, by carefully integrating an extensive collection of literature data, researchers have developed large size EMT GRNs, from which the dynamic features of cellular state transitions can be identified. However, EMT is not a standalone process, but tightly associated with other biological processes, including, but not limited to, intercellular communication by Notch signaling pathway, cell motility, metabolism, cell proliferation, stem cell differentiation, and immunity.

### 2.3 EMT circuits coupled with other processes

Many efforts have been made to understand the role of Notch signaling pathway in regulating EMT-induced cell motility during normal development and cancer metastasis [43–45]. These led to modeling the coupling between the Notch–Delta signaling pathway and the EMT core circuit, which provided a mechanistic understanding of how the hybrid E/M state induces and maintains the metastatic cellular clusters via intercellular communication [46, 47]. Cohen et al. developed a 30-node Boolean network to study synergistic combination of Notch overexpression and p53 deletion in cancer metastasis [39].

Furthermore, several studies modeled the coupling between metabolic pathways, EMT, and metastasis. Yu et al. constructed a coarse-grained 4-node network model of two metabolic pathways glycolysis and oxidative phosphorylation (OXPHOS) to study the interplay between the two pathways and the gene regulation of metabolic plasticity [48], with the implication of their roles in cancer metastasis. Jia et al. further extended the network with detailed interactions among regulatory genes and metabolites; their modeling predictions of metabolic plasticity were experimentally validated using several cancer cell lines [49]. Subsequently, Kang et al. [50] modeled, with a landscape approach, a 16-node metabolism-EMT-metastasis network that integrates metabolism circuit [48], EMT core circuit [15, 51, 52], and metastasis circuit [53]. Some recent mathematical models focused on mechanical interactions to understand the gene regulation of cells losing cellular cohesion during EMT [54–56].

In summary, researchers employed the bottom-up approaches to construct EMT GRNs of different sizes, whose mathematical modeling elucidated the regulatory mechanism of EMT and its coupling with other pathways. Despite its success in modeling EMT GRN, the bottom-up approach is typically limited by the following factors: (1) literature synthesis can be quite tedious and time consuming; (2) because of the involvement of significant manual curation, it is not straightforward to reproduce literature-based GRNs; (3) there may not be sufficient literature data to investigate the EMT process in a particular biological context. Additional information on the bottom-up approach can also be found in some recent reviews [14, 57–59].

## 3 THE TOP-DOWN APPROACH

Another approach to model EMT GRNs is a top-down approach of constructing networks from bioinformatics algorithms using genome-wide sequencing data, such as transcriptomics data (bulk and single-cell RNA-seq) and epigenomics data (Assay for Transposase-Accessible Chromatin [ATAC-seq], chromatin immunoprecipitation [ChIP-seq]). These genome-wide data can be utilized to unbiasedly infer transcription factor (TF)-target relations based on statistical association (such as correlation, mutual information and regression) and their occurrence in experimental and literature databases (such as TF-target databases and TF binding motif database) [60–64]. One advantage of these top-down approaches is that they help tailor the GRN to the dataset of interest by emphasizing interactions reflected in the (epi)genomics data [65–68]. Compared to bottom-up approaches, top-down approaches also streamline the network construction process, making GRN modeling analysis more efficient and reproducible. On the other hand, the top-down approaches usually lead to large GRNs, therefore the network construction is more liable to overfitting and is more adversely affected by sparsity and noise in the data [69]. Moreover, most bioinformatics-based GRNs are not evaluated according to their ability to capture network dynamics. Below, we will summarize the basic components of top-down methodologies and describe some of their recent applications to EMT.

### 3.1 Bioinformatics algorithms for GRN construction

The increasing availability of multiple omics studies represents an opportunity to develop a new, more cohesive model of EMT regulation. Indeed, a rich resource of transcriptomics and epigenomics data are publicly available on the study of EMT GRNs, as summarized in Table 1 [5, 65, 67, 68, 70–92]. Many bioinformatics algorithms have been developed to construct GRNs from these resources [60–64, 69]. In recent years, scRNA-seq data have become particularly popular for GRN construction, mainly because of rapid advances in genomic technology and computational methodologies. In the study of EMT, single-cell transcriptomics can be especially important for the discovery of cell phenotypic heterogeneity and the dynamical transitions between cellular states [5, 36, 93]. Thus, network construction using scRNA-seq is more likely to generate networks capturing these features of EMT.

Although different GRN construction methods have their own approaches, they typically deploy common steps of bioinformatic analyses as part of their algorithms. In the following, we will take scRNA-seq data as an example to illustrate these bioinformatic techniques. First, the raw sequencing data need to be aligned to a reference genome and converted to gene expression counts [94]. Second, the count data are normalized by gene length and library size and log-transformed [95]. The gene expression data must also be processed to correct batch effects and/or remove cells/genes with low counts [96]. Third, having preprocessed the data, one can perform certain downstream analyses such as (1) visualizing the transcriptomic landscape via dimensional reduction [97] (principal component analysis [PCA] [98], t-stochastic neighbor embedding [t-SNE] [99], uniform manifold approximation and projection [UMAP]) [100]; (2) identifying distinct cellular phenotypes by gene expression clustering [101] (k-means, hierarchical clustering, etc.); (3) identifying important genes, pathways, or gene ontology (GO) terms that are distinct between the cellular phenotypes using differential expression analysis [102] (limma [103], DESeq2) [104] and gene-set-based enrichment analysis (GSEA [105, 106], GSA [107], GSVA) [108]; (4) inferring pseudo-time [109] in the case that time series data are unavailable.

Finally, there is a growing suite of software packages designed to analyze single-cell sequencing data, some of which have provided functionality for GRN construction. For example, a package termed single-cell regulatory network inference and clustering (SCENIC) [60] works by identifying highly correlated modules of genes and cross-referencing these with TF binding motifs from the cisTarget database [110]. Another method, Dynamic Regulatory Events Miner (DREM) [111, 112], can be used to construct dynamic GRNs from time series data by identifying timepoints where coexpressed genes diverge, using GO terms to annotate the biological mechanisms behind each split. Other tools like Cicero [113] are used to construct GRNs from chromatin accessibility data instead of RNA-seq by identifying regulatory elements coaccessible with gene promoters [63, 114]. Recently, Pratapa et al. [69] developed a framework entitled BEELINE to evaluate the quality of network construction algorithms using scRNA-seq data based on criteria including accuracy, scalability, and the level of detail they output. The authors benchmarked 12 network construction algorithms with several simulated and experimental datasets with known network topologies and identified PIDC [115], GENIE3 [116], and GrnBoost2 [117] as having the best overall performance. They also found that inaccurate pseudo-time labels can be detrimental, and that many methods infer edges where only an indirect relationship exists, creating unintended feedforward structures. In summary, computational methods for GRN construction are growing in number and sophistication. Although sparsity and noise remain challenging obstacles, these tools provide an accessible framework to infer regulatory links from transcriptomics and epigenomic data.

### 3.2 EMT GRN Construction

An example that encapsulates the top-down approach to EMT modeling is a 2016 work from Chang et al. [118] where the authors uncovered synergistic behavior of three EMT regulators: ETS2, HNF4A, and JUNB. The authors first performed RNA-seq on TGF-*β* treated A549 cells over a period of 96 h, identifying three distinct, sequentially activated groups of genes, which they associate to E, hybrid, and M phenotypes. GO terms confirmed these findings, as the gene sets enriched in the hybrid and M cells were increasingly related to cell motility and adhesion. Interestingly, however, certain canonical EMT markers like SNAI1/2, TWIST1/2, and ZEB1/2 did not appear to be key regulators in this dataset. Hypothesizing that important transcription factors (TFs) may have been as yet unknown, the authors performed binding motif enrichment for putative EMT TFs based on the time series data. They then examined ChIP-seq data, finding additional evidence that the candidate TFs indeed bind to the locations of hundreds of differentially expressed genes in the experiment. Finally, the authors applied DREM to the time series data to pinpoint temporal changes in regulation. Major splitting points were identified at the 6-h and 48-h timepoints and included regulatory changes among the previously indicated TFs, possibly reflecting transitions from E to hybrid and hybrid to M states, respectively. The approach adopted by Chang et al. permitted a thorough and contextual analysis of EMT in A549 cells, despite the apparent lack of activity among many canonical EMT factors. By examining EMT on the basis of multi-gene signatures and quantified trends in gene expression, top-down approaches thus stand to improve the accuracy and applicability of EMT GRNs.

Top-down approaches can also reveal context specific (i.e., dependent on tissue type, time, input signal, etc.) EMT regulatory mechanisms, by applying inference tools to transcriptomic data or epigenetic sequencing like ATAC-seq [65, 67, 68]. Cook and Vanderhyden recently examined four cancer cell lines undergoing EMT induced via three different signaling conditions, using time series measurements to observe distinct trajectories and patterns of TF activity according to the context of the EMT [65]. Only a small number of the genes that responded to the three signals were shared across all conditions, demonstrating how much context can influence the EMT regulatory network. In another study, Wouters et al. [92] constructed GRNs based on SOX10 KD-induced EMT in melanoma at various timepoints by taking the consensus results of SCENIC over 100 runs, supplementing SCENIC’s use of TF binding motifs with ATAC-seq chromatin accessibility information. The authors found that much of the data could be explained by a spectrum of melanocytic, intermediate, and mesenchymal-like phenotypes, noting that the consensus GRN for intermediate states was a stable mixture of regulations from both extreme phenotypes. The authors leveraged software tools and public repositories to map the EMT trajectory in melanoma with a high degree of detail. Although algorithmic GRN inference has far to go, in the case of EMT many of these tools have proven capable of recapitulating known findings and identifying new and/or cell type-specific regulatory interactions.

Top-down, bioinformatic-based approaches to model EMT have proven useful in characterizing the transcriptomic landscape of EMT and even in algorithmically constructing GRNs. This approach permits a thorough analysis of the phenotypic space, with single-cell sequencing providing the necessary granularity to construct GRNs that accurately reflect the observed distribution of cell states. Additionally, computational tools can make GRN construction more efficient, scalable, and reproducible. However, despite many available tools and datasets, constructing highly accurate EMT GRNs from bioinformatics results alone has proven challenging. Feature measurements are often noisy, impacting the accuracy of downstream analyses. Additionally, it remains challenging to distinguish, directly from the data, different types of regulation (e.g., methylation, transcriptional, translational) and identify key regulators. As a result, automatically constructed networks are prone to contain redundant structures or spurious links between genes that may be in shared modules, but do not directly interact [64, 69]. Although top-down analyses are especially useful for examining phenotypic heterogeneity, algorithmically constructed GRNs can benefit greatly from additional validation or optimization. Mathematical network modeling is thus a natural progression from bioinformatics approaches; inferred GRNs can be integrated into dynamical models and interactions iteratively refined by examining their dynamical properties in comparison to experimentally observed behaviors.

## 4 COMBINED TOP-DOWN AND BOTTOM-UP APPROACH

To overcome the limitations of both the bottom-up and top-down approaches, some recent studies seek to combine mathematical modeling with bioinformatic network construction. This approach offers a number of potential advantages as follows. First, EMT transition paths and key regulators can depend on the system in which they occur, so networks pulled together from general databases and literature search may not be relevant to a particular system of interest [65, 78, 119]. In these scenarios, bioinformatic analysis on associated transcriptomics and epigenetic data can contribute to incorporate context-specific regulatory relationships [35, 66, 91]. Second, the combined approach can improve the quality of the GRNs constructed by bioinformatics methods, as mathematical modeling can evaluate whether the GRNs can capture the gene expression dynamics of the biological process. Ideally, this approach combines the features of simplicity and predictivity from the bottom-up approach and the features of reproducibility and robustness to literature bias/errors from the top-down approach. Examples of studies with combined approaches and their corresponding methodology are summarized in Table 2 [35, 36, 42, 66, 91, 120–123].

One approach that combines top-down and bottom-up methodologies is to first construct a large network, then identify subnetworks that describe EMT in different contexts. Khan et al. [35] constructed an 879-node, 2278-edge network for the E2F TF family based on extensive manual review of published literature, characterizing its role in processes including EMT, cell cycle, DNA repair, and apoptosis. This large network, while comprehensive, would be unwieldy to investigate in the context of specific tumor types. Therefore, the authors identified subnetworks that described EMT in breast and bladder cancer by identifying the most important network structures in each type. Motifs were ranked on multiple metrics including involvement in cancer pathways, fold-change between invasive and noninvasive specimens, and topological properties. They conducted Boolean simulations on 41- and 35-node subnetworks for bladder and breast cancer respectively, finding unique combinatorial EMT-inducing signals, each associated with more aggressive tumors of their respective tissue type in The Cancer Genome Atlas (TCGA) cohort data [35]. By ranking key network motifs according to multiple factors including transcriptomics data and topological properties, Khan et al. facilitate the construction of GRNs that are highly representative of specific biological conditions.

Udyavar et al. [36] describes another integrated study examining subtypes of small cell lung cancer (SCLC). Using ARACNE, a large network was generated and subsequently filtered by cross-referencing with multiple binding site and databases including ENCODE [124], TRANSFAC [125], EnrichR [126], and PubMed [127]. Subsequent Boolean simulations of this GRN predicted the expected NE and ML subtypes, but failed to capture a hybrid phenotype present in tumor samples. In a follow-up work by Wooten et al., a Boolean modeling approach called BooleaBayes was developed that infers the probability that each node is ON or OFF based on gene expression patterns of similar states, allowing more nuanced relationships than traditional Boolean modeling. Conducting in silico perturbations with BooleaBayes revealed likely stabilizers and destabilizers of each SCLC subtype, suggesting targets for therapies aimed at driving SCLC tumors from an aggressive subtype to a more tractable one [91]. These studies together illustrate the complementary nature of top-down with bottom-up methods: the initial top-down GRN alone, while in agreement with experimental data, failed to accurately recapitulate the observed phenotypic landscape in a simple mathematical model. Integrating systematic validation against literature and binding motifs improved the model’s predictive capabilities, with a more sophisticated mathematical model finally bringing the simulated results into close agreement with observed data.

Another strategy for combining top-down and bottom-up methods is to begin from a well-supported, manually curated core topology and augment it with a context-specific set of interactions such that modeling can approximate the observed bioinformatic data. Kohar et al., integrated a literature-based GRN with networks from squamous cell carcinoma and modeled it with RACIPE. The GRN simulations accurately depict the E, M, and hybrid states observed in the gene expression data, with further improvements in accuracy when gene expression noise was implemented in the modeling [42]. The integration of SCC-specific topologies and well-established EMT motifs improved the agreement between the steady states predicted by RACIPE and those observed in the data. Furthermore, some efforts have been made to systematically generate the context specific interaction set while preserving the fundamental behavior of the core circuit. Ramirez et al. combined a core EMT topology with new interactions found by applying SCENIC to time series scRNA-seq data comparing EMT in four cell lines as induced by three different signaling conditions [65]. Considering each experimental condition separately, Ramirez et al. constructed, simulated, and refined context-specific GRNs by testing an ensemble of network construction parameters and finding the optimal GRN for each case. The primary criteria for inclusion in the network were (1) a correlation between regulator and target gene in the expression data for the relevant cell line, and (2) proximity to the core topology, as interactions were added incrementally moving outward from the core (both upstream and downstream). Although the resulting GRNs varied between experimental conditions, they included several highly conserved genes, suggesting that EMT may be governed by a small set of master regulators with flexible roles [66]. An iterative, optimization-based approach to network construction is expected to greatly improve the accuracy of EMT modeling studies.

This third category of studies, wherein networks are constructed using both broadly supported evidence from the literature and context-specific interactions from bioinformatics, then subsequently simulated with mathematical models, represents an evolution in quality and reproducibility in EMT modeling research. Integrated studies can not only identify genes of interest or infer individual regulatory links but can make testable predictions about complex dynamical behaviors and master regulators, facilitating the discovery of clinical tools targeting EMT. On the other hand, integrated methodologies are early in development, with few established best practices or formalized workflows, and some critical limitations. One obstacle is the breadth of background knowledge required to properly integrate bioinformatics with more traditional modeling approaches. Moreover, combined approaches tend to involve larger GRNs, which can be both more difficult to validate experimentally and more computationally expensive to model.

## 5 DISCUSSION AND PERSPECTIVES

One of the major challenges in biology is to understand the gene regulatory mechanisms that determine the decision making of cellular state transitions. In this paper, we reviewed three different types of computational systems biology approaches for modeling EMT-associated GRNs. The first approach relies on literature data for network construction. Being the gold-standard methodology in the field of systems biology, the literature-based method utilizes network interactions derived from dedicated experimental studies in biochemistry, cell biology and genetics, most of them having high accuracy. Thus, the literature-based approach results in high-quality GRNs to recapitulate existing biology. However, it may not work well in the case where biology literature is incomplete and/or inconsistent (e.g., in the studies of cancer biology) [128]. It is also tedious, time consuming, and error-prone to construct a large GRN. Note that, although most literature-based GRN modeling provides a list of experimental evidences for GRN regulatory interactions, little is usually given to describe how GRNs were constructed step by step, making most of the literature synthesis steps irreproducible. The literature-based approach also does not work well to study GRNs specific to a particular experimental condition, disease type, and subjects of certain genetic background.

The second approach constructs GRNs using bioinformatics analysis on genomics data from a specific experiment. Being a mainstream approach in current genomics and computational biology studies, it utilizes statistical analysis on gene expression data (e.g., bulk RNA-seq, scRNA-seq) and/or epigenetics data (such as ATAC-seq, Hi-C) to identify potential gene regulatory interactions. In some studies, literature data were also integrated, but in the format of a database containing curated gene regulatory interactions, biochemical/metabolic reactions, or from in silico prediction based on transcription factor binding sites. This approach addresses certain issues from the former approach—in particular, it allows modeling for a specific biological context and potentially identifying novel interactions. Because of the top-down approach, it usually results in GRNs of larger size. However, it has been shown that the current network construction methods are still insufficient to construct high-quality GRNs [69]. One of the issues is network redundancy. As many regulators and interactions between them are redundant in a biological system to achieve robustness, it is hard to reverse engineer the correct interactions back directly from data such as gene expression. Moreover, although bioinformatics is an ideal tool to identify regulators and biological pathways, it is seldom evaluated whether a GRN constructed through bioinformatics can operate as a dynamic biological system. This becomes a critical problem, particularly in the studies of cellular state transition like EMT, as network dynamics is an essential component of the biological process.

The third approach combines both the bottom-up and top-down approaches to construct GRNs. Conceptually, this is a better way to address the issues of the previous two approaches. By incorporating genomics data and literature databases together with mathematical modeling, one can model context specific GRNs that capture the dynamical behavior of cellular state transitions. We have seen recent studies on EMT GRN modeling with such a strategy, yet it remains a quite challenging task owing to the following reasons. First, systems biology modeling and bioinformatics belong to two very distinct research disciplines, making it difficult for researchers to grasp sufficient knowledge to be experienced in both research fields. Second, building a high-quality GRN model remains difficult with the combined approach. It is not uncommon that important regulators and/or signaling pathways, which are well known in the literature, cannot be identified from the genome-wide data directly. Thus, it is important to have better databases containing high-quality regulatory interactions and signaling pathways. More sophisticated computational algorithms are also needed to accurately identify context specific regulatory interactions, for example, by integrating a variety of types of genomics data and biological evidence. Third, as another central component of this approach, a powerful mathematical modeling algorithm is needed to capture the dynamics of a large GRN in an unbiased and efficient way. In particular, the ensemble-based approach in some recent studies seems to be a promising technique [37, 42, 66]. Last but not least, experimental validation is crucial for better GRN modeling. As the constructed GRNs can be especially large, it is important to devise validations, such as high-throughput gene perturbation, that allow to evaluate the quality of a large system. In summary, we foresee that the combined top-down and bottom-up approach, although still in its infancy, could be a powerful tool in the future GRN modeling studies on EMT and also other biological cellular state transitions.

## Figures and Tables

**TABLE 1:** Published EMT transcriptomics and epigenomics datasets

Assay type	Assay name	Experiment	Description	Reference
Transcriptomic	Microarray	GSE121372	Human HPMCs treated with TGF-b1	Han et al.,2019 [73]
		GSE88762	EMT in mouse tumor-initiating cells	Latil et al., 2017 [76]
		GSE87877	EMT in mouse tumor-initiating cells	Latil et al., 2017 [76]
		GSE53923	Ovol2 in EMT in mouse terminal end buds	Watanabe et al., 2014 [89]
		GSE53175	EMT in a breast cancer primary culture	Minafra et al., 2014 [79]
		GSE39368	Molecular subtypes of head and neck cancer	Walter et al., 2013 [86]
		GSE42373	TGF-*β*/TNF-*α*-treated A549 spheroids	Wamsley et al., 2015 [87]
		GSE17708	Time course of A549 cells treated with TGF-*β*	Sartor et al., 2010 [82]
		GSE17538	Four experiments, colon cancer in humans and mice	Smith et al., 2010 [83]
		GSE9691	E-cadherin loss in human epithelial cells	Onder et al., 2008 [80]; Taube et al., 2010 [84]
	RNA-seq	GSE145850	MCF10A cells treated with TGF-*β*	Johnson et al., 2020 [75]
		GSE124843	Perturbing TGF-*β* and ZEB1 in MCF10A	Watanabe et al., 2019 [90]
		GSE110585	Hybrid EMT states in mouse tumor tissues	Pastushenko et al., 2018 [81]
		GSE70741	hESC differentiation into hepatoctyes	Li et al., 2017 [77]
		GSE88989	EMT in mouse tumor-initiating cells	Latil et al., 2017 [76]
		GSE59987	Hypoxia-induced EMT in human cancer cells	Tsai et al., 2014 [85]; Wang et al., 2020 [88]
	scRNA-seq	GSE147405	Time course scRNA-seq in human cancer cell lines	Cook et al., 2020 [65]
		GSE134432	scRNA-seq and ATAC-seq of melanoma tissues	Wouters et al., 2020 [92]
		GSE135893	EMT in pulmonary fibrosis and healthy lungs	Habermann et al., 2020 [72]
		GSE114687	EMT in MCF10A and HuMEC cells	McFaline-Figueroa et al., 2019 [78]
		GSE137749	Two triple knockout SCLC mouse models	Wooten et al., 2019 [91]
		GSE110357	Hybrid EMT states in mouse tumor tissues	Pastushenko et al., 2018 [81]
		GSE114397	TGF-*β*-induced EMT in HMLE cells	van Dijk et al., 2018 [71]
		GSE100037	Mouse bone marrow lymphoid progenitors	Herman et al., 2018 [74]
		GSE87038	EMT in mouse organogenesis	Dong et al., 2018 [5]
		GSE103322	Head and neck cancer	Puram et al., 2017 [67]
Epigenomic	ChIP-seq	GSE80218	Hypoxia-regulated EMT in FADU cell line	Wang et al., 2020 [88]
		GSE61198	EMT in normal and cancerous mouse stem cells	Ye et al., 2015 [68]
		GSM1303689	Ovol2 ChIP-seq in mouse terminal end buds	Watanabe et al., 2014 [89]
		GSE42374	TGF-*β*/TNF-*α*-treated A549 spheroids	Cieslik et al., 2013 [70]
	ATAC-seq	GSE145851	TGF-*β*-induced EMT in MCF10A cells	Johnson et al., 2020 [75]
		GSE134432	scRNA-seq and ATAC-seq of melanoma tissues	Wouters et al., 2020 [92]
		GSE114397	TGF-*β*-induced EMT in HMLE cells	van Dijk et al., 2018 [71]
		GSE110584	Hybrid EMT states in mouse tumor tissues	Pastushenko et al., 2018 [81]
		GSE70474	EMT in mouse tumor-initiating cells	Latil et al., 2017 [76]
	hMeDIP-seq	GSE59989	Hypoxia-induced EMT in human cancer cells	Tsai et al., 2014 [85]

**TABLE 2:** Combined-approach study methodologies

Reference	Subject	Modeling	Bioinformatics	Integration
Examples of GRN modeling of EMT				
Khan et al., 2017 [35]	E2F-mediated EMT in cancer	Boolean network simulations and in silico perturbations	E2F family interactions curated from TRANSFAC, STRING, HPRD, MiRTarBase; >98% validated by domain experts	GRNs for breast and bladder cancer constructed by ranking global network motifs by (1) topological properties, (2) agreement with gene expression in target datasets, (3) agreement with KEGG cancer pathways
Udyavar et al., 2017 [36]; Wooten et al., 2019 [91]	EMT in SCLC	Developed BooleaBayes, a Boolean network modeling framework that can also estimate probabilities	Clustering, weighted gene coexpression network analysis (WGCNA), and GRN inference with ARACNE filtered with TF-target databases, literature review	Boolean network modeling to predict multiple SCLC subtypes and subtype-specific master regulators
Kohar and Lu, 2018 [42]	EMT in SCC	Ensemble ODE-based simulations with RACIPE and stochastic noise	Incorporated GRNs from a previous study on Epcam+ and Epcam− cells using RNA-seq and ATAC-seq	Combination of manually curated core EMT network with SCC-specific networks from previous genome-wide study
Ramirez et al., 2020 [66]	EMT in cancer	Ensemble ODE-based simulations with RACIPE	SCENIC used to infer GRNs for each dataset and identify conserved and context-specific interactions	Iterative GRN construction and SCENIC parameter optimization by comparing simulated and experimental data
Sha et al., 2020 [123]	EMT in cancer and embryogenesis	Stochastic ODE-based multiscale simulation of a core EMT circuit	QuanTC is developed, which identifies clusters, marker genes, and transition genes from scRNA-seq data	QuanTC applied to multiple EMT datasets to validate the behaviors predicted by the model
Examples of GRN modeling of other processes				
Moignard et al., 2015 [120]	Mouse hematopoiesis	Boolean network modeling	Single-cell quantitative reverse transcription polymerase chain reaction (qRT-PCR) on ~40 genes; Developed single-cell network synthesis (SCNS) toolkit to construct Boolean networks from discretized expression data	Using SCNS, a GRN was constructed to identify key regulators, which were later validated experimentally
Dunn et al., 2014 [122]; Dunn et al., 2019 [121]	mESCs	Abstract Boolean network (ABN) modeling—ensemble Boolean networks based on experimental constraints	Initial coexpression network from microarray and RNA-seq data, qRT-PCR and clonal assays with siRNA to test model predictions	Iteratively refined a meta-model of multiple Boolean networks by experimentally validating model predictions
